# Shoelace Wound Closure for the Management of Fracture-Related Fasciotomy Wounds

**DOI:** 10.5402/2012/528382

**Published:** 2012-09-19

**Authors:** Abdelsalam Eid, Mohamed Elsoufy

**Affiliations:** Department of Orthopaedic Surgery, Faculty of Medicine, Zagazig University, 5 Mahfouz Street from Ahmed Ismail Street, Zagazig 44511, Egypt

## Abstract

*Background*. Compartment syndrome is a serious complication that might occur following fractures. The treatment of choice is emergent fasciotomy of all the involved muscle compartments to lower the compartment pressure. The classic management of fasciotomy wounds was split thickness skin graft. *Patients and Methods*. Seventeen patients with fracture-related compartment syndrome were managed by fasciotomy in the Orthopaedic Casualty Unit of our university hospital. The fractures included four femoral fractures and 13 fractures of the tibia and fibula. *Results*. All fasciotomy wounds healed eventually. Wound closure occurred from the corners inward. The skin closure was obtained at an overall average of 4.2 tightening sessions (range 3–7). Fracture healing occurred at an average of 15.4 weeks (range 12 to 22 weeks). No major complications were encountered in this series. *Conclusion*. Closure of fasciotomy wounds by dermatotraction could be performed in a staged fashion, using inexpensive equipment readily available in any standard operating room, until skin was approximated enough to heal either through delayed primary closure or secondary healing.

## 1. Introduction

Compartment syndrome is a serious complication that might occur following fractures. Untreated, it would cause serious damage to the nervous and muscular structures of the involved compartment(s), which might lead to serious and permanent functional deficit of the involved limb. The treatment of choice is emergent fasciotomy of all the involved muscle compartments to lower the compartment pressure [1–4]. Fasciotomy wounds can seldom be closed primarily because muscles under tension bulge through the wound making primary closure not feasible. The classic management of fasciotomy wounds was split thickness skin graft. This however led to an unsightly appearance as well as insensate area of skin over the graft [1–4]. Since no skin loss has occurred with the fasciotomy, and owing to the skin's ability to relax when under stress (creep), several authors [4–14] considered the use of skin stretching techniques to gradually or acutely close fasciotomy wounds. This process has been called by some authors dermatotration [6, 7]. To achieve this, some used specialized and costly equipment [4–8]. Our hypothesis was that closure of fasciotomy wounds by dermatotraction could be performed in a staged fashion, using inexpensive equipment readily available in any standard operating room, until skin was approximated enough to heal either through delayed primary closure or secondary healing.

## 2. Patients and Methods

**Figure 1:**
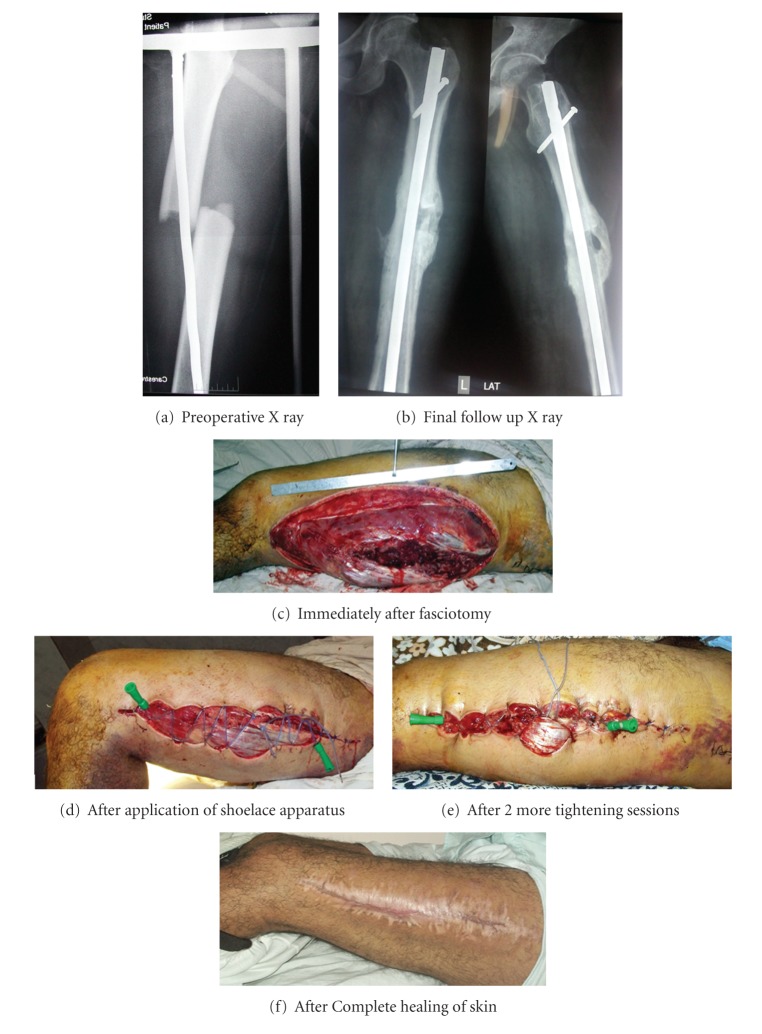
26-year-old male. Car accident. Fracture midshaft femur.

**Figure 2:**
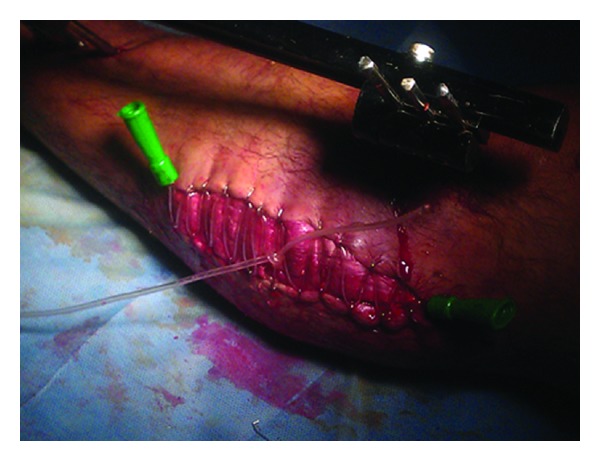
19-year-old male. Fracture tibia and fibula. After fasciotomy and fixation by external fixator.

Between June 2006 and May 2010, 17 patients with fracture-related compartment syndrome were managed by fasciotomy in the Orthopaedic Casualty Unit of our university hospital (Table 1). All work was conducted in accordance with the Declaration of Helsinki (1964). Institutional review board approval was obtained. The fractures included four femoral fractures and 13 fractures of the tibia and fibula. The inclusion criteria were closed fractures, no concomitant skin loss, fracture-related compartment syndrome, and fasciotomy within 36 hours. Exclusion criteria were open fractures, skin loss, non-fracture-related compartment syndrome, and delay of presentation beyond 36 hours. Compartment syndrome diagnosis was made depending on the clinical examination and measurement of compartment pressure by a senior surgeon. In three femoral fractures, and two tibia and fibula fractures, the compartment syndrome was diagnosed during or immediately after the operation for internal fixation, as the limb swelling increased and became quite tense after the insertion of intramedullary nails. In the remaining twelve cases where the diagnosis of compartment was made on admission, unreamed interlocking nail was used for stabilization of the femoral fracture, while the tibial fractures were fixed by monolateral external fixators in the same setting as the fasciotomy. In three cases of femoral fractures, a midlateral fasciotomy was performed to decompress the extensor and flexor compartments only (Figure 1), while in the fourth case of femoral fracture, two fasciotomy wounds were performed laterally and medially to decompress the extensor, flexor, and the adductor compartment. In eight of both the bone fractures, a single-incision fasciotomy was used to decompress the four compartments of the leg (Figure 2), while in the remaining five cases, a two-incision (medial and lateral) fasciotomy was performed. The total number of fasciotomy wounds treated in this study thus was 23 wounds. The fasciotomy was performed at an average of 21.1 hours (range 12–36 hours) following the injury. The patients were 13 males and four females. The average age was 23.3 years (range 16 to 35 years). All injuries were related to high energy trauma (8 motorcycle accidents, 5 pedestrian accidents, 4 falls from a height). 

Daily dressing was applied for the fasciotomy wound and the wound was reinspected after 48 hours. This delay was deemed necessary to identify any damage to the muscles and deep structures that might compromise the cleanliness of the wound and prevent its closure. In three cases, the fasciotomy wound had to be debrided in the operative theatre 2 to 3 times before it was judged clean enough to begin wound closure. 

### 2.1. Surgical Technique

A paediatric urinary catheter was anchored to the skin edge beginning from one corner of the wound using skin staples. Then the catheter was passed from one edge of the wound to the other in an alternating fashion, each time being fixed to the skin using skin staples applied perpendicular to the wound edge. In five rather large wounds, two catheters were used starting from the two corners of the wound to meet at the centre of the wound. After the catheter(s) had been passed allover the wound, tightening was performed beginning at the end of the wound and proceeding towards the other end. In case of the five wounds where two catheters were used, tightening began from both ends of the wound and proceeded centrally. Tightening was done by pulling on the catheter and passing the slack through the staples one at a time until the maximal approximation of the edges without undue skin tension was obtained. Then the catheter was knotted on itself in small wounds, or tied to the other catheter over the centre of the wound in large wounds. A wet dressing was applied and the wound was left for two days to allow the skin to accommodate the approximation that has been obtained. Retightening was done in the operative theatre with only a sedative every two to three days. 

## 3. Followup

The patients were discharged from the hospital after wound closure was obtained. They came back for follow upat 1, 3, and 6 months postoperatively. Afterwards they came for clinical and radiological followup every 6 months. The average followup was 17.1 months (range 12 to 24).

## 4. Results

All fasciotomy wounds healed eventually. Wound closure occurred from the corners inward. The skin closure was obtained at an overall average of 4.2 tightening sessions (range 3–7). In twelve cases, as the skin at the corners was approximated, it became possible to place sutures or staples across the wound thus allowing delayed primary closure after an average of 3.8 tightening sessions (3–5). While in 5 cases, the wounds were allowed to close by granulation tissue. All fractures initially fixed by intramedullary nail (IMN) were managed definitively by the same implant. As regards, the 11 fractures was treated initially by an external fixator, 7 fractures were eventually fixed by IMN, while 4 were fixed by minimally invasive plate osteosynthesis (MIPO). 

Fracture healing occurred at an average of 15.4 weeks (range 12 to 22 weeks). In 8 cases, additional procedures were performed during the follow-up period, either to assist healing (2 dynamizations and 4 bone grafts) or to remove the implant after healing because of deep infection (2 cases).

## 5. Complications

No major complications (e.g., vascular compromise, amputations) were encountered in this series. In five cases, persistent wound discharge indicated the presence of previously undetected deep soft tissue necrosis. The patient was taken to the operating theater for removal of the shoelace apparatus and further debridement of necrotic tissue (once in 4 cases, and twice in 1 case). When the local wound conditions improved, the shoelace apparatus was reapplied in the OR under general anaesthesia, and tightening sessions were carried out again as explained before. These wounds were allowed to heal by secondary intention.

## 6. Discussion

Dermatotraction utilizes the skin's characteristics of stress relaxation (creep). The results of dermatotraction have been shown to be superior to split thickness skin graft as it has better cosmetic appearance, provides sensate skin, and avoids donor site morbidity. 

 In our series, all fasciotomy wounds closed at an overall average of 4.2 tightening sessions. The shoelace apparatus used for closure consisted of one or two paediatric urinary catheter, plus surgical skin staples, which in our setting cost US $ 10–12. The material is readily available in any standard operating theatre, making this procedure useful for countries with limited resources. This supports our hypothesis that closure of fasciotomy wounds by dermatotraction could be performed in a staged fashion, using inexpensive equipment readily available in any standard operating room, until skin was approximated enough to heal either through delayed primary closure or secondary healing.

Numerous devices have been utilized to obtain skin closure by dermatotraction (Table 2). Barnea et al. [4] used the Wisebands device, Hirshowitz et al. [5] used the Sure Closure device, Janzing and Broos [6] used the Marburger skin approximation system, Taylor et al. [8] used skin anchors, and Govaert and Van Helden [9] used Ty-Raps. 

However, the use is limited by availability and expense. This is especially important in a developing country with limited resources of the healthcare system.

Marek et al. [7] and Chiverton and Redden [10] suggested obtaining complete wound closure by dermatotraction in a single session. This is often not possible due to the size of the wound and the excessive amount of traction necessary to close large wounds, which may lead to failure of the apparatus, or blanching and impairment of skin vascularity. But even in small wounds it may end in failure because sometimes deep muscle damage and necrosis do not appear immediately on presentation leading to persistent wound discharge and failure of wound closure, and in addition, there is the potential risk of reelevating compartment pressure. In this series, we did not attempt to obtain complete wound closure in a single session in any of our patients. Skin closure was obtained at an overall average of 4.2 tightening sessions (range 3–7), which allowed us to reexamine the wounds in the OR and redo debridement when necessary.

An important limitation of this technique is the absence of a built-in monitoring system or other safety mechanism to monitor compartment pressure or skin tension. This is left to rely totally on the surgeon's experience and judgment. This is why the technique should be performed only under the close direct supervision of an experienced surgeon.

## 7. Conclusion

Closure of fasciotomy wounds by dermatotraction could be performed in a staged fashion, using inexpensive equipment readily available in any standard operating room, until skin was approximated enough to heal either through delayed primary closure or secondary healing.

## Figures and Tables

**Table 1:** Patients' data.

Patient number	Sex	Age (years)	Fracture	Delay before fasciotomy (hours)	Number of fasciotomy wounds	Method of final closure	Number of tightening sessions	Further debridement after shoelace	Duration till complete wound healing (weeks)	Initial method of fixation	Definitive method of fixation	Duration to fracture healing (weeks)	Additional procedures	Followup (months)
1	M	19	BB	20	1	1ry	3	No	3	Ex Fix	IMN	14		12
2	M	21	BB	16	1	1ry	3	No	3	IMN	IMN	16	Dynamization	18
3	M	27	Fem	31	1	2ry	5	1	4	IMN	IMN	14	Removal	18
4	F	17	BB	19	1	1ry	4	No	3	Ex Fix	MIPO	16		16
5	M	17	BB	24	1	1ry	4	No	3	Ex Fix	IMN	14	Bone graft	24
6	M	23	BB	22	2	1ry	5	No	4	Ex Fix	MIPO	16		16
7	M	30	Fem	30	1	2ry	5	1	4	IMN	IMN	14	Bone graft	12
8	M	29	BB	12	2	1ry	3	No	3	Ex Fix	IMN	16		18
9	M	31	BB	14	1	1ry	4	No	4	Ex Fix	IMN	14		20
10	M	27	BB	12	2	1ry	3	No	3	Ex Fix	IMN	12		12
11	F	18	BB	15	2	1ry	5	No	4	Ex Fix	MIPO	14	Bone graft	22
12	M	26	Fem	34	2	2ry	7	2	6	IMN	IMN	22	Removal	24
13	F	35	BB	29	1	2ry	5	1	5	Ex Fix	IMN	16		16
14	M	21	Fem	21	1	1ry	4	No	4	IMN	MIPO	16		18
15	M	20	BB	33	2	2ry	5	1	5	IMN	IMN	18	Dynamization	20
16	F	16	BB	15	1	1ry	4	No	4	Ex Fix	IMN	16	Bone graft	12
17	M	19	BB	12	1	1ry	3	No	3	Ex Fix	IMN	14		12

Average		23.3		21.1			4.2		3.8			15.4		17.1

F: female, M: male, Fem: femur, BB: both bones of the leg, 1ry: primary, 2ry: secondary, Ex Fix: external fixator, IMN: intramedullary nail, MIPO: minimally invasive plate osteosynthesis.

**Table 2:** Comparison between different wound closure devices.

Authors	Devices	Advantages	Disadvantages
Barnea et al. [4]	Wisebands device	(i) Tension feedback control mechanism to safeguard against excessive skin tensioning	(i) Not readily available
			(ii) Expensive
Hirshowitz et al. [5]	Sure Closure device	(i) Can measure the tension across	(i) Not readily available
		the wound edges	(ii) Expensive
Janzing and Broos [6]	Marburger skin approximation system		(i) Not readily available
			(ii) Expensive
Taylor et al. [8]	Skin anchors	(i) Anchors placed 1 cm away from the wound edge to prevent circulatory compromise at the skin edge	(i) Not readily available
		(ii) Evenly distributed force over the full length of the wound	(ii) Expensive
Govaert and van Helden [9]	Ty-Raps		(i) Not readily available
This study	Paediatric urinary catheters + skin staples	(i) Readily available	(i) Point loading on the staples may lead to their failure
		(ii) Inexpensive	(ii) No safe mechanism against excess tension
